# The Role of Molecular Imaging in Precision Oncology

**DOI:** 10.2967/jnumed.124.268036

**Published:** 2024-11

**Authors:** James R. Bading, Joanne E. Mortimer

**TO THE EDITOR:** Two articles in the March 2024 issue of *The Journal of Nuclear Medicine* (1*,*^2^) repeat the often-made assertion that molecular imaging can be used to visualize tumor expression of molecular targets for antitumor therapy. However, the relationship between uptake and target expression may be confounded by a variety of factors, including delivery of the radiopharmaceutical from the intravascular injection site to tumor cells (3).

More importantly, when the imaging agent accurately traces the biodistribution of the treatment drug, molecular imaging enables measurement of relative drug dose to tumor. In that capacity, molecular imaging is likely to be a better predictor of treatment response and patient benefit than is tumor expression of the drug target. Compelling evidence for that assertion is provided by PET studies with ^89^Zr- and ^64^Cu-labeled trastuzumab in patients with histopathologically HER2-positive advanced breast cancer preceding treatment with the antibody–drug conjugate trastuzumab emtansine (4*,*^5^).

## DISCLOSURE

Joanne Mortimer is a consultant for Daiichi Sankyo, Astra-Zeneca, Novartis, and GE HealthCare. She also receives research funding from AstraZeneca. No other potential conflict of interest relevant to this article was reported.

## Notes

**James R. Bading, Joanne E. Mortimer***

**City of Hope Comprehensive Cancer Center Duarte, California*

*E-mail: jmortimer@coh.org*
